# Supplementary material for: A Likelihood Approach for Real-Time Calibration of Stochastic Compartmental Epidemic Models
Source: PLoS Comput Biol. 2017 Jan 17;13(1):e1005257. doi: 10.1371/journal.pcbi.1005257 (PMC5240920; doi:10.1371/journal.pcbi.1005257)
Supplement: S1 File — (TAR.GZ) [file pcbi.1005257.s014.tar.gz › HSPH_Online-SI-Revision/PF08/n100-severe/n100-severe_PF2_table0.pdf]

1	newly 3 weeks spec	5129.	6548.	{6232., 6749.}	27.6662	0.105896	newly 3 weeks cumul	17951.	20546.6	{19523., 21047.}	14.4593	0.0584955	newly 5 weeks spec	3152.	5211.13	{4920., 5337.}	65.3277	0.21817	newly 5 weeks cumul	25181.	31698.3	{30076., 32424.}	25.8818	0.0998125	newly 50 weeks cumul	33334.	56185.3	{53614., 57032.}	68.5526	0.226627
2	newly 3 weeks spec	3271.	8731.63	{8577., 8901.}	166.941	0.426366	newly 3 weeks cumul	16291.	30070.2	{29858., 30350.}	84.5814	0.266177	newly 5 weeks spec	952.	5671.67	{5559., 5802.}	495.764	0.775003	newly 5 weeks cumul	19055.	42881.3	{42540., 43518.}	125.04	0.352245	newly 57 weeks cumul	20150.	61183.5	{60678., 62238.}	203.64	0.48234
3	newly 3 weeks spec	3370.	9328.34	{9198., 9441.}	176.805	0.442152	newly 3 weeks cumul	19722.	31605.9	{31380., 31795.}	60.257	0.204811	newly 5 weeks spec	834.	6134.2	{6007., 6252.}	635.516	0.866539	newly 5 weeks cumul	22331.	45421.5	{45126., 45654.}	103.401	0.308349	newly 58 weeks cumul	23238.	65174.7	{64914., 65399.}	180.466	0.447879
4	newly 3 weeks spec	5397.	8367.6	{8256., 8492.}	55.0417	0.190416	newly 3 weeks cumul	21357.	28372.3	{28092., 28670.}	32.8478	0.12334	newly 5 weeks spec	2735.	5614.06	{5478., 5764.}	105.267	0.312236	newly 5 weeks cumul	27895.	40954.7	{40644., 41253.}	46.8174	0.166769	newly 56 weeks cumul	32739.	59911.	{59026., 60657.}	82.9957	0.262416
5	newly 3 weeks spec	4803.	5942.33	{5690., 6338.}	23.7212	0.0921075	newly 3 weeks cumul	16297.	19092.7	{18486., 20287.}	17.1544	0.0684774	newly 5 weeks spec	3233.	4590.15	{4409., 4911.}	41.978	0.151858	newly 5 weeks cumul	23516.	28980.2	{27987., 31085.}	23.236	0.0904664	newly 50 weeks cumul	34577.	50154.7	{48344., 53309.}	45.0522	0.161257
6	newly 3 weeks spec	6383.	8719.72	{8544., 8917.}	36.6085	0.135422	newly 3 weeks cumul	23683.	28182.7	{27751., 28616.}	18.9997	0.0755134	newly 5 weeks spec	2924.	6216.92	{6055., 6345.}	112.617	0.327513	newly 5 weeks cumul	31005.	41893.8	{41357., 42533.}	35.1195	0.130688	newly 56 weeks cumul	36697.	64155.2	{63134., 65026.}	74.8242	0.24257
7	newly 3 weeks spec	5854.	9226.04	{9091., 9373.}	57.6023	0.197534	newly 3 weeks cumul	26186.	30870.6	{30656., 31099.}	17.8898	0.0714696	newly 5 weeks spec	2014.	6158.67	{6052., 6270.}	205.793	0.485382	newly 5 weeks cumul	31770.	44704.1	{44381., 45071.}	40.7117	0.148323	newly 57 weeks cumul	34576.	64962.8	{64396., 65621.}	87.8842	0.273879
8	newly 3 weeks spec	4856.	7349.57	{7190., 7538.}	51.3503	0.179911	newly 3 weeks cumul	17949.	23092.1	{22758., 23740.}	28.6542	0.109372	newly 5 weeks spec	2677.	5626.78	{5478., 5772.}	110.19	0.322517	newly 5 weeks cumul	24358.	35273.6	{34824., 36099.}	44.8132	0.160768	newly 53 weeks cumul	30079.	57894.3	{57108., 58551.}	92.4741	0.284351
9	newly 3 weeks spec	4469.	10725.7	{10579., 10830.}	140.003	0.380195	newly 3 weeks cumul	27073.	31010.5	{30828., 31176.}	14.5439	0.0589672	newly 5 weeks spec	876.	8267.73	{8138., 8389.}	843.805	0.974851	newly 5 weeks cumul	30073.	49017.1	{48797., 49224.}	62.9937	0.212168	newly 58 weeks cumul	30570.	78524.8	{78360., 78685.}	156.869	0.409711
10	newly 3 weeks spec	5097.	5352.82	{5230., 5454.}	5.01903	0.0212063	newly 3 weeks cumul	20668.	14931.5	{14785., 15095.}	27.7554	0.141209	newly 5 weeks spec	2025.	5347.88	{5232., 5465.}	164.093	0.421695	newly 5 weeks cumul	25993.	25740.6	{25474., 25959.}	1.00489	0.00439524	newly 51 weeks cumul	29196.	63389.7	{63056., 63694.}	117.118	0.336692
11	newly 3 weeks spec	4623.	9216.12	{9043., 9379.}	99.3537	0.299585	newly 3 weeks cumul	21770.	31353.8	{31052., 31687.}	44.0231	0.158419	newly 5 weeks spec	1559.	6014.09	{5851., 6166.}	285.766	0.586233	newly 5 weeks cumul	26128.	44937.2	{44475., 45474.}	71.9888	0.235482	newly 57 weeks cumul	28027.	64373.	{63608., 65193.}	129.682	0.361104
12	newly 3 weeks spec	5357.	5681.79	{5580., 5764.}	6.06291	0.0255322	newly 3 weeks cumul	18503.	16616.2	{16456., 16772.}	10.1974	0.0467216	newly 5 weeks spec	3340.	5208.26	{5119., 5318.}	55.9359	0.192888	newly 5 weeks cumul	26203.	27356.4	{27160., 27563.}	4.40167	0.0187	newly 50 weeks cumul	35015.	59145.1	{58842., 59420.}	68.9137	0.227661
13	newly 3 weeks spec	5099.	6275.39	{5999., 6506.}	23.071	0.0899545	newly 3 weeks cumul	17831.	20006.8	{19235., 20621.}	12.2022	0.049833	newly 5 weeks spec	3072.	4895.63	{4629., 5108.}	59.363	0.202105	newly 5 weeks cumul	24961.	30520.8	{29303., 31390.}	22.2741	0.0871651	newly 50 weeks cumul	33743.	53017.4	{50434., 54925.}	57.1212	0.196006
14	newly 3 weeks spec	4962.	5992.37	{5776., 6198.}	20.7652	0.0817436	newly 3 weeks cumul	15685.	18388.	{17793., 19057.}	17.2331	0.0689073	newly 5 weeks spec	3640.	5002.24	{4802., 5135.}	37.4242	0.137731	newly 5 weeks cumul	23643.	28968.9	{28069., 29851.}	22.5265	0.0880671	newly 50 weeks cumul	36706.	54542.4	{53188., 54847.}	48.5927	0.171723
15	newly 3 weeks spec	4360.	7082.78	{6957., 7194.}	62.4491	0.210681	newly 3 weeks cumul	17059.	20966.4	{20737., 21163.}	22.905	0.0895558	newly 5 weeks spec	2303.	6095.96	{5971., 6226.}	164.696	0.422693	newly 5 weeks cumul	22470.	33762.2	{33386., 34055.}	50.2545	0.176815	newly 52 weeks cumul	26744.	64142.7	{63844., 64453.}	139.84	0.379918
16	newly 3 weeks spec	5728.	6810.84	{6693., 6934.}	18.9043	0.0751547	newly 3 weeks cumul	18259.	20255.4	{20026., 20498.}	10.934	0.045045	newly 5 weeks spec	4036.	5846.67	{5720., 5963.}	44.863	0.160906	newly 5 weeks cumul	27272.	32528.6	{32210., 32894.}	19.2746	0.0765325	newly 51 weeks cumul	38769.	62762.5	{62422., 63134.}	61.8883	0.209211
17	newly 3 weeks spec	4479.	9724.6	{9570., 9860.}	117.115	0.336656	newly 3 weeks cumul	20396.	31195.8	{30703., 31637.}	52.9507	0.18452	newly 5 weeks spec	1516.	6775.57	{6635., 6919.}	346.937	0.650181	newly 5 weeks cumul	24628.	46271.8	{45599., 46786.}	87.8828	0.273866	newly 57 weeks cumul	26798.	69179.8	{68311., 69808.}	158.153	0.41186
18	newly 3 weeks spec	4189.	8655.9	{8334., 9019.}	106.634	0.314986	newly 3 weeks cumul	17056.	28116.7	{27223., 29197.}	64.8493	0.216882	newly 5 weeks spec	1958.	5936.88	{5565., 6447.}	203.211	0.481133	newly 5 weeks cumul	22027.	41368.8	{39963., 43342.}	87.8093	0.273485	newly 55 weeks cumul	25613.	60232.	{56919., 66171.}	135.162	0.370625
19	newly 3 weeks spec	3570.	7779.49	{7552., 7967.}	117.913	0.338213	newly 3 weeks cumul	17052.	27098.5	{26404., 27541.}	58.9171	0.201107	newly 5 weeks spec	1298.	5104.51	{4950., 5208.}	293.26	0.594585	newly 5 weeks cumul	20704.	38605.7	{37693., 39181.}	86.4648	0.270546	newly 55 weeks cumul	22668.	55637.5	{54385., 56311.}	145.445	0.38992
20	newly 3 weeks spec	4499.	7561.94	{7457., 7668.}	68.0805	0.225489	newly 3 weeks cumul	19401.	22700.	{22532., 22857.}	17.0042	0.0681939	newly 5 weeks spec	1849.	6258.18	{6148., 6378.}	238.463	0.529468	newly 5 weeks cumul	24272.	35973.4	{35741., 36177.}	48.2096	0.170872	newly 53 weeks cumul	27031.	64749.2	{64526., 64987.}	139.537	0.37937
21	newly 3 weeks spec	5237.	9223.37	{9098., 9355.}	76.1193	0.24578	newly 3 weeks cumul	23703.	30795.1	{30565., 30994.}	29.9205	0.11367	newly 5 weeks spec	1742.	6179.91	{6050., 6289.}	254.759	0.549881	newly 5 weeks cumul	28600.	44657.5	{44368., 44985.}	56.1452	0.193522	newly 57 weeks cumul	30632.	65058.9	{64582., 65685.}	112.389	0.327123
22	newly 3 weeks spec	5006.	7842.33	{7672., 7977.}	56.6586	0.194904	newly 3 weeks cumul	21806.	21855.3	{21513., 22137.}	0.893791	0.00387629	newly 5 weeks spec	1880.	7105.04	{6971., 7213.}	277.928	0.577368	newly 5 weeks cumul	26703.	36635.5	{36095., 37016.}	37.1963	0.137323	newly 55 weeks cumul	29097.	69361.1	{68269., 69943.}	138.379	0.377249
23	newly 3 weeks spec	1987.	10340.	{10183., 10457.}	420.38	0.716298	newly 3 weeks cumul	17807.	33221.1	{32967., 33459.}	86.5621	0.270817	newly 5 weeks spec	308.	7105.53	{6982., 7221.}	2206.99	1.36301	newly 5 weeks cumul	18940.	49089.1	{48797., 49339.}	159.182	0.413601	newly 59 weeks cumul	19129.	72556.7	{72228., 72774.}	279.302	0.578983
24	newly 3 weeks spec	4945.	10366.6	{10215., 10516.}	109.637	0.321443	newly 3 weeks cumul	26504.	32577.3	{32380., 32795.}	22.9145	0.0895974	newly 5 weeks spec	1389.	7306.37	{7172., 7413.}	426.017	0.720967	newly 5 weeks cumul	30352.	48791.1	{48539., 49016.}	60.7508	0.20615	newly 60 weeks cumul	31658.	73308.4	{73044., 73531.}	131.564	0.364669
25	newly 3 weeks spec	3439.	10456.6	{10262., 10610.}	204.06	0.482923	newly 3 weeks cumul	21402.	32670.	{32038., 33033.}	52.6492	0.183666	newly 5 weeks spec	698.	7407.69	{7260., 7510.}	961.274	1.02578	newly 5 weeks cumul	23735.	49092.5	{48221., 49570.}	106.836	0.315601	newly 60 weeks cumul	24404.	74061.9	{73029., 74597.}	203.483	0.482119
26	newly 3 weeks spec	5656.	7587.26	{7479., 7689.}	34.1453	0.127547	newly 3 weeks cumul	23609.	22145.9	{21960., 22310.}	6.19717	0.0277919	newly 5 weeks spec	2372.	6465.52	{6331., 6592.}	172.577	0.435434	newly 5 weeks cumul	29758.	35777.	{35485., 36012.}	20.2266	0.0799932	newly 54 weeks cumul	33191.	64625.	{64338., 64894.}	94.7064	0.289378
27	newly 3 weeks spec	4510.	8874.19	{8769., 8989.}	96.767	0.293928	newly 3 weeks cumul	21137.	28803.6	{28617., 28961.}	36.2708	0.134397	newly 5 weeks spec	1681.	6250.53	{6144., 6356.}	271.834	0.57031	newly 5 weeks cumul	25596.	42641.2	{42427., 42827.}	66.5934	0.221655	newly 55 weeks cumul	27946.	64719.4	{64548., 64958.}	131.587	0.364713
28	newly 3 weeks spec	3624.	7290.26	{6811., 7767.}	101.166	0.303014	newly 3 weeks cumul	14491.	23621.6	{22168., 25001.}	63.009	0.21175	newly 5 weeks spec	2108.	5172.11	{4726., 5566.}	145.356	0.388952	newly 5 weeks cumul	19406.	35070.9	{32821., 37285.}	80.7219	0.256524	newly 54 weeks cumul	24332.	52701.4	{48567., 56245.}	116.593	0.334894
29	newly 3 weeks spec	4912.	8078.63	{7937., 8247.}	64.4672	0.216032	newly 3 weeks cumul	20248.	27868.8	{27409., 28269.}	37.6373	0.138708	newly 5 weeks spec	2132.	5322.13	{5174., 5457.}	149.631	0.397179	newly 5 weeks cumul	25828.	39833.2	{39252., 40380.}	54.2248	0.18813	newly 55 weeks cumul	29513.	57516.2	{56651., 58357.}	94.8843	0.289745
30	newly 3 weeks spec	4348.	5906.9	{5793., 6037.}	35.8533	0.133024	newly 3 weeks cumul	16097.	17398.1	{17190., 17556.}	8.08287	0.0337405	newly 5 weeks spec	2202.	5260.16	{5148., 5368.}	138.881	0.378126	newly 5 weeks cumul	21394.	28336.4	{28090., 28576.}	32.4501	0.12204	newly 51 weeks cumul	26261.	57791.6	{57462., 58110.}	120.066	0.342549
31	newly 3 weeks spec	1715.	10404.5	{10268., 10532.}	506.676	0.782937	newly 3 weeks cumul	14771.	32642.1	{32456., 32816.}	120.988	0.344364	newly 5 weeks spec	342.	7329.12	{7219., 7434.}	2043.02	1.331	newly 5 weeks cumul	15873.	48906.2	{48653., 49107.}	208.109	0.488703	newly 60 weeks cumul	16071.	73566.	{73371., 73751.}	357.756	0.660633
32	newly 3 weeks spec	4733.	10002.5	{9892., 10137.}	111.336	0.324954	newly 3 weeks cumul	23783.	31913.4	{31723., 32067.}	34.1856	0.127702	newly 5 weeks spec	1187.	6974.96	{6850., 7087.}	487.612	0.769055	newly 5 weeks cumul	27483.	47427.5	{47200., 47602.}	72.5701	0.236963	newly 58 weeks cumul	28771.	70852.	{70660., 71023.}	146.262	0.391396
33	newly 3 weeks spec	3999.	8910.96	{8804., 9021.}	122.83	0.347944	newly 3 weeks cumul	20824.	30885.5	{30658., 31095.}	48.317	0.171184	newly 5 weeks spec	1142.	5748.36	{5647., 5847.}	403.359	0.701836	newly 5 weeks cumul	24133.	43902.7	{43651., 44184.}	81.9196	0.259874	newly 58 weeks cumul	25686.	62261.2	{61924., 62613.}	142.394	0.384517
34	newly 3 weeks spec	4878.	5109.69	{4986., 5204.}	4.74969	0.0200943	newly 3 weeks cumul	19185.	14268.7	{14094., 14432.}	25.626	0.128596	newly 5 weeks spec	2525.	5140.97	{5036., 5250.}	103.603	0.308738	newly 5 weeks cumul	25181.	24618.3	{24366., 24826.}	2.23454	0.00982622	newly 50 weeks cumul	30277.	62145.2	{61798., 62448.}	105.255	0.31229
35	newly 3 weeks spec	3516.	10693.5	{10555., 10815.}	204.138	0.483048	newly 3 weeks cumul	21757.	32866.7	{32591., 33089.}	51.0625	0.17915	newly 5 weeks spec	674.	7691.81	{7556., 7809.}	1041.22	1.05734	newly 5 weeks cumul	24081.	49902.1	{49648., 50139.}	107.226	0.316441	newly 59 weeks cumul	24720.	76037.7	{75809., 76294.}	207.596	0.487979
36	newly 3 weeks spec	6059.	10013.	{9869., 10130.}	65.2578	0.21814	newly 3 weeks cumul	27874.	31532.4	{31368., 31712.}	13.1247	0.0535531	newly 5 weeks spec	2044.	7096.11	{6982., 7204.}	247.168	0.540509	newly 5 weeks cumul	33550.	47255.9	{47034., 47538.}	40.852	0.14876	newly 57 weeks cumul	35952.	71438.1	{71213., 71769.}	98.7041	0.298205
37	newly 3 weeks spec	2491.	9754.56	{9612., 9883.}	291.592	0.592811	newly 3 weeks cumul	16769.	31968.5	{31780., 32103.}	90.6407	0.280212	newly 5 weeks spec	544.	6608.58	{6514., 6695.}	1114.81	1.08449	newly 5 weeks cumul	18580.	46764.8	{46571., 46969.}	151.694	0.400871	newly 59 weeks cumul	19084.	68590.9	{68409., 68756.}	259.415	0.555596
38	newly 3 weeks spec	4614.	9176.06	{9054., 9311.}	98.8743	0.298554	newly 3 weeks cumul	23415.	31393.	{31159., 31602.}	34.072	0.127332	newly 5 weeks spec	1497.	5974.84	{5841., 6070.}	299.121	0.601058	newly 5 weeks cumul	27574.	44893.7	{44613., 45131.}	62.8117	0.211681	newly 57 weeks cumul	29436.	64069.	{63747., 64319.}	117.655	0.337767
39	newly 3 weeks spec	3711.	7096.71	{6796., 7533.}	91.2344	0.281296	newly 3 weeks cumul	14691.	23342.3	{22459., 24298.}	58.8887	0.200909	newly 5 weeks spec	1875.	4914.99	{4637., 5530.}	162.133	0.417736	newly 5 weeks cumul	19116.	34252.8	{33012., 36183.}	79.184	0.253063	newly 54 weeks cumul	22836.	50739.9	{47627., 58399.}	122.193	0.345701
40	newly 3 weeks spec	4019.	9288.21	{9096., 9464.}	131.107	0.36375	newly 3 weeks cumul	16800.	29953.4	{29392., 30465.}	78.294	0.251091	newly 5 weeks spec	1644.	6482.86	{6300., 6662.}	294.335	0.595641	newly 5 weeks cumul	20945.	44370.9	{43503., 44943.}	111.845	0.325976	newly 57 weeks cumul	23590.	66153.3	{64515., 67356.}	180.429	0.447644
41	newly 3 weeks spec	3584.	9649.14	{9540., 9747.}	169.228	0.430101	newly 3 weeks cumul	19699.	32227.6	{32024., 32424.}	63.6001	0.213778	newly 5 weeks spec	987.	6403.25	{6274., 6504.}	548.759	0.812042	newly 5 weeks cumul	22587.	46642.2	{46392., 46851.}	106.5	0.314918	newly 59 weeks cumul	23519.	67474.8	{67249., 67654.}	186.895	0.457722
42	newly 3 weeks spec	4951.	7180.19	{7047., 7309.}	45.025	0.161408	newly 3 weeks cumul	19337.	21368.9	{21172., 21586.}	10.5077	0.0433813	newly 5 weeks spec	2604.	6131.18	{6000., 6268.}	135.452	0.371849	newly 5 weeks cumul	25605.	34269.8	{33955., 34550.}	33.8401	0.126576	newly 52 weeks cumul	30663.	64397.9	{64021., 64714.}	110.018	0.322253
43	newly 3 weeks spec	3668.	6824.42	{6654., 6974.}	86.0529	0.269525	newly 3 weeks cumul	13660.	20821.5	{20325., 21406.}	52.427	0.182961	newly 5 weeks spec	2047.	5632.23	{5520., 5770.}	175.146	0.43948	newly 5 weeks cumul	18398.	32768.2	{32128., 33403.}	78.1073	0.250615	newly 51 weeks cumul	23176.	60202.3	{59660., 60762.}	159.762	0.414539
44	newly 3 weeks spec	5502.	10107.8	{9967., 10234.}	83.7117	0.264116	newly 3 weeks cumul	26971.	32044.7	{31840., 32262.}	18.8117	0.0748542	newly 5 weeks spec	1807.	7093.19	{6984., 7200.}	292.54	0.593856	newly 5 weeks cumul	31936.	47815.1	{47616., 48006.}	49.7217	0.175282	newly 58 weeks cumul	33949.	71695.9	{71515., 71966.}	111.187	0.324666
45	newly 3 weeks spec	5745.	8969.38	{8800., 9148.}	56.125	0.193421	newly 3 weeks cumul	23088.	29062.3	{28679., 29698.}	25.8761	0.0999121	newly 5 weeks spec	2549.	6300.86	{6178., 6432.}	147.189	0.392973	newly 5 weeks cumul	29573.	43021.5	{42630., 43722.}	45.4754	0.162767	newly 56 weeks cumul	33764.	65012.5	{64461., 65606.}	92.5496	0.284528
46	newly 3 weeks spec	5945.	7580.54	{7451., 7694.}	27.5112	0.105512	newly 3 weeks cumul	25387.	21065.7	{20850., 21247.}	17.0219	0.0810475	newly 5 weeks spec	2372.	6948.4	{6816., 7081.}	192.934	0.466716	newly 5 weeks cumul	31718.	35478.6	{35055., 35813.}	11.8564	0.0486482	newly 55 weeks cumul	35057.	68063.7	{67760., 68359.}	94.1516	0.288138
47	newly 3 weeks spec	4772.	6381.74	{6209., 6587.}	33.733	0.126121	newly 3 weeks cumul	17003.	20453.7	{19910., 20891.}	20.2949	0.0801649	newly 5 weeks spec	2927.	4912.99	{4770., 5074.}	67.8507	0.224776	newly 5 weeks cumul	23808.	31048.7	{30446., 31620.}	30.4129	0.115253	newly 51 weeks cumul	31769.	53161.3	{51955., 54376.}	67.337	0.223461
48	newly 3 weeks spec	5735.	8525.87	{8368., 8707.}	48.6638	0.172144	newly 3 weeks cumul	21931.	28438.1	{28074., 28876.}	29.6706	0.112799	newly 5 weeks spec	2847.	5815.39	{5671., 5972.}	104.264	0.310092	newly 5 weeks cumul	29108.	41396.1	{41045., 41991.}	42.2155	0.152919	newly 56 weeks cumul	34186.	61233.1	{60259., 62501.}	79.1174	0.253088
49	newly 3 weeks spec	3521.	11039.3	{10900., 11164.}	213.528	0.496256	newly 3 weeks cumul	23225.	33446.8	{33214., 33676.}	44.012	0.158393	newly 5 weeks spec	685.	8003.69	{7869., 8122.}	1068.42	1.06757	newly 5 weeks cumul	25469.	51107.4	{50821., 51350.}	100.665	0.302468	newly 60 weeks cumul	25996.	78203.8	{77926., 78486.}	200.83	0.47832
50	newly 3 weeks spec	2974.	9840.14	{9703., 9970.}	230.872	0.519636	newly 3 weeks cumul	18646.	32648.9	{32455., 32813.}	75.0984	0.243278	newly 5 weeks spec	685.	6556.39	{6456., 6662.}	857.137	0.980941	newly 5 weeks cumul	20804.	47400.9	{47210., 47585.}	127.845	0.357637	newly 59 weeks cumul	21381.	68693.1	{68426., 68900.}	221.281	0.506883
